# T cell activation markers CD38 and HLA-DR indicative of non-seroconversion in anti-CD20-treated patients with multiple sclerosis following SARS-CoV-2 mRNA vaccination

**DOI:** 10.1136/jnnp-2023-332224

**Published:** 2024-03-28

**Authors:** Niels J M Verstegen, Ruth R Hagen, Christine Kreher, Lisan H Kuijper, Jet van den Dijssel, Thomas Ashhurst, Laura Y L Kummer, Virginia Palomares Cabeza, Maurice Steenhuis, Mariël C Duurland, Rivka de Jongh, C Ellen van der Schoot, Veronique A L Konijn, Erik Mul, Katherine Kedzierska, Koos P J van Dam, Eileen W Stalman, Laura Boekel, Gertjan Wolbink, Sander W Tas, Joep Killestein, Theo Rispens, Luuk Wieske, Taco W Kuijpers, Filip Eftimov, Zoé L E van Kempen, S Marieke van Ham, Anja ten Brinke, Carolien E van de Sandt

**Affiliations:** 1 Department of Immunopathology, Sanquin Research and Landsteiner Laboratory, Amsterdam UMC, University of Amsterdam, Amsterdam, The Netherlands; 2 Department of Hematopoiesis, Sanquin Research and Landsteiner Laboratory, Amsterdam UMC, University of Amsterdam, Amsterdam, The Netherlands; 3 Department of Experimental Immunohematology, Sanquin Research and Landsteiner Laboratory, Amsterdam UMC, University of Amsterdam, Amsterdam, The Netherlands; 4 Sydney Cytometry Core Research Facility, Charles Perkins Centre, Centenary Institute, and The University of Sydney, Sydney, NSW, Australia; 5 School of Medical Sciences, Faculty of Medicine and Health, The University of Sydney, Sydney, NSW, Australia; 6 Department of Neurology and Neurophysiology, Amsterdam Neuroscience, Amsterdam UMC, location AMC, University of Amsterdam, Amsterdam, The Netherlands; 7 Research Facilities, Sanquin Research and Landsteiner Laboratory, Amsterdam UMC, University of Amsterdam, Amsterdam, The Netherlands; 8 Department of Microbiology and Immunology, University of Melbourne at the Peter Doherty Institute for Infection and Immunity, Melbourne, VIC, Australia; 9 Global Station for Zoonosis Control, Global Institution for Collaborative Research and Education (GI-CoRE), Hokkaido University, Sapporo, Japan; 10 Department of Rheumatology, Amsterdam Rheumatology and Immunology Center location Reade, Amsterdam, The Netherlands; 11 Department of Clinical Immunology and Rheumatology, Amsterdam Rheumatology and Immunology Center, Amsterdam UMC, University of Amsterdam, Amsterdam, The Netherlands; 12 Department of Neurology, Amsterdam UMC, Amsterdam UMC, Vrije Universiteit, Amsterdam, The Netherlands; 13 Department of Clinical Neurophysiology, St Antonius Hospital, Nieuwegein, The Netherlands; 14 Department of Pediatric Immunology, Rheumatology and Infectious Disease, Amsterdam UMC, Location AMC, University of Amsterdam, Amsterdam, The Netherlands; 15 Swammerdam Institute for Life Sciences, University of Amsterdam, Amsterdam, The Netherlands

**Keywords:** MULTIPLE SCLEROSIS, COVID-19

## Abstract

**Background:**

Messenger RNA (mRNA) vaccines provide robust protection against SARS-CoV-2 in healthy individuals. However, immunity after vaccination of patients with multiple sclerosis (MS) treated with ocrelizumab (OCR), a B cell-depleting anti-CD20 monoclonal antibody, is not yet fully understood.

**Methods:**

In this study, deep immune profiling techniques were employed to investigate the immune response induced by SARS-CoV-2 mRNA vaccines in untreated patients with MS (n=21), OCR-treated patients with MS (n=57) and healthy individuals (n=30).

**Results:**

Among OCR-treated patients with MS, 63% did not produce detectable levels of antibodies (non-seroconverted), and those who did have lower spike receptor-binding domain-specific IgG responses compared with healthy individuals and untreated patients with MS. Before vaccination, no discernible immunological differences were observed between non-seroconverted and seroconverted OCR-treated patients with MS. However, non-seroconverted patients received overall more OCR infusions, had shorter intervals since their last OCR infusion and displayed higher OCR serum concentrations at the time of their initial vaccination. Following two vaccinations, non-seroconverted patients displayed smaller B cell compartments but instead exhibited more robust activation of general CD4^+^ and CD8^+^ T cell compartments, as indicated by upregulation of CD38 and HLA-DR surface expression, when compared with seroconverted patients.

**Conclusion:**

These findings highlight the importance of optimising treatment regimens when scheduling SARS-CoV-2 vaccination for OCR-treated patients with MS to maximise their humoral and cellular immune responses. This study provides valuable insights for optimising vaccination strategies in OCR-treated patients with MS, including the identification of CD38 and HLA-DR as potential markers to explore vaccine efficacy in non-seroconverting OCR-treated patients with MS.

**WHAT IS ALREADY KNOWN ON THIS TOPIC**

- Previous studies indicated that patients with multiple sclerosis (MS) treated with anti-CD20 have reduced antibody formation and increased T cell-mediated immune responses upon SARS-CoV-2 vaccination, associated with an increased risk of breakthrough infections in patients with MS who did not develop antibodies (non-seroconverted). However, there is a lack of comprehensive studies that examine the immunological differences between seroconverted and non-seroconverted patients with MS treated with anti-CD20, as well as the absence of biomarkers to detect cellular immunity in non-seroconverted patients.

**WHAT THIS STUDY ADDS**

- Through our comprehensive immunological analysis of patients with MS treated with anti-CD20, we found that prior to SARS-CoV-2 vaccination, both seroconverting and non-seroconverting patients exhibited similar B and T cell profiles, but the vaccine only enhanced the B cell compartment in seroconverted patients, whereas larger T cell responses, marked by increased levels of CD38 and HLA-DR, were observed in non-seroconverting patients. We could directly link those differences to the number of anti-CD20 infusions, the time since the last treatment and the anti-CD20 concentration in the blood.

**HOW THIS STUDY MIGHT AFFECT RESEARCH, PRACTICE OR POLICY**

- These findings emphasize the significance of incorporating treatment frequency, time since last treatment, and anti-CD20 levels in the blood into the scheduling of SARS-CoV-2 vaccination for MS patients to optimize both their humoral and cellular immune responses, with the latter being detectable through measurements of CD38 and HLA-DR expression on circulating T cell subsets.

## Introduction

Multiple sclerosis (MS) is a chronic immune-mediated inflammatory disorder characterised by the immune system attacking the protective myelin sheath surrounding nerve fibres in the central nervous system. This disrupts nerve signal transmission and causes various neurological symptoms.^1^ Ocrelizumab (OCR), an anti-CD20 antibody, effectively treats MS by depleting CD20-positive B cells, reducing inflammation and disease progression.^2^

During the COVID-19 pandemic, widespread vaccination efforts have been crucial in mitigating the severity and transmission of SARS-CoV-2. This significance was amplified for patients undergoing anti-CD20 therapies, as they were previously associated with a more severe progression of COVID-19 before vaccination.^3^ Although certain patients undergoing anti-CD20 treatments can generate detectable levels of antibodies (seroconversion),^4–9^ the majority of patients receiving anti-CD20 therapies like OCR or rituximab exhibit suboptimal antibody responses to SARS-CoV-2 vaccination, potentially compromising their level of protection.^4–8 10–14^ Indeed, breakthrough infections are more frequently reported in anti-CD20-treated SARS-CoV-2-vaccinated patients with MS who did not seroconvert, although severe infections remain rare.^15^ We must gain a better understanding of the immunological distinctions between seroconverted and non-seroconverted OCR-treated patients with MS, particularly from a clinical perspective to predict which patients are at risk of not seroconverting and identify immunological markers to indicate if they are potentially protected from severe disease by other arms of the immune system. Our previous investigations indicated that OCR serum concentration or B cell counts at the time of vaccination were predictive of the success of SARS-CoV-2 receptor-binding domain (RBD)-specific antibody formation in OCR-treated patients with MS.^4 16^ In the present study, we aimed to explore the immune dynamics following pre-SARS-CoV-2 and post-SARS-CoV-2 vaccination to identify immunological markers to better categorise patients and tailor vaccination strategies to improve immune responses and strengthen protection against SARS-CoV-2.

## Materials and methods

### Study participants and design

As part of a national prospective longitudinal study (the target-to-B! COVID-19 Study) investigating the humoral response following SARS-CoV-2 vaccination, individuals aged over 18 years were recruited, including healthy donors, patients with relapsing-remitting MS without systemic immunomodulatory treatment and patients with MS treated with OCR. The study was registered with the Dutch Trial Register (ID NL8900).

**Figure 1:**
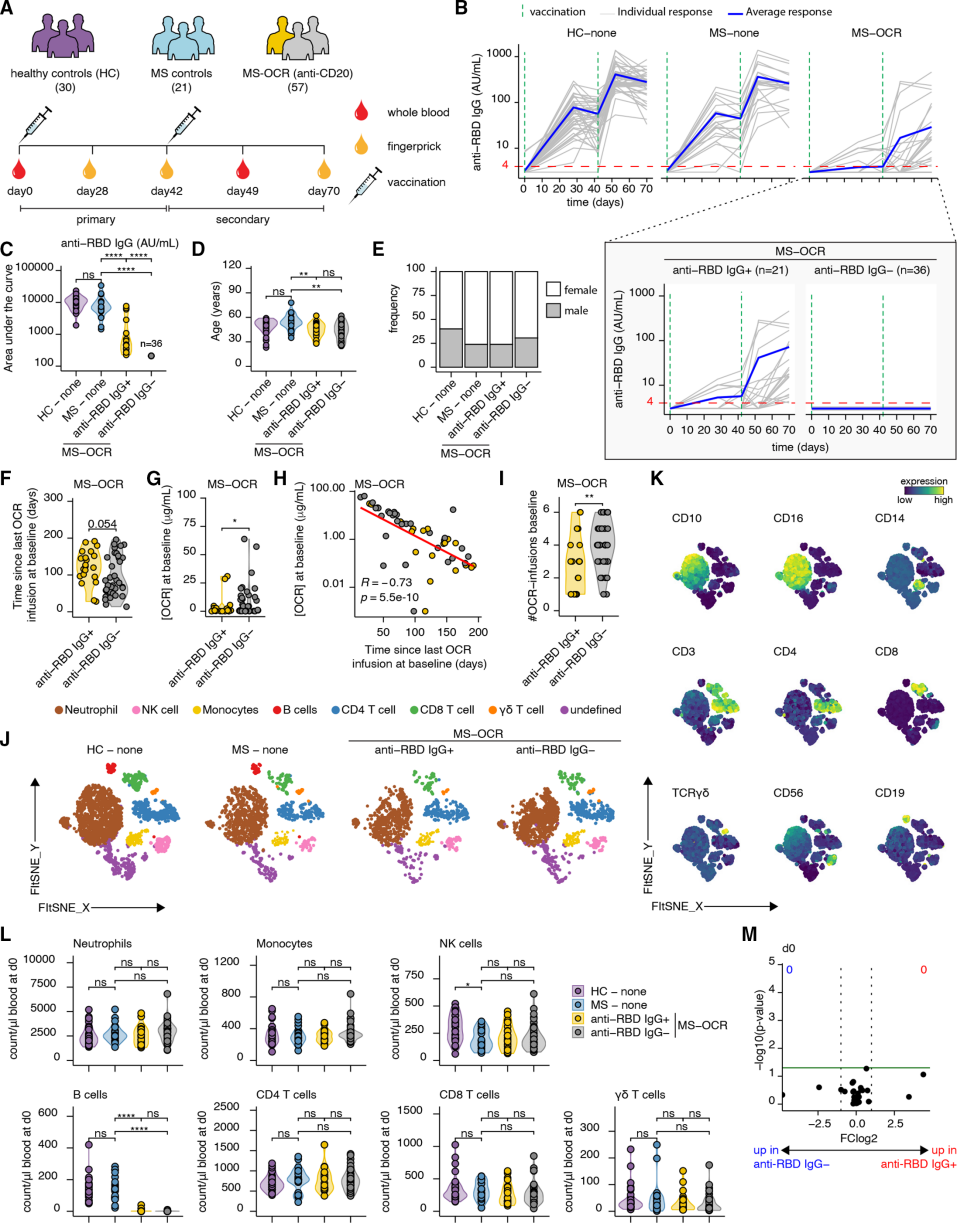
Circulating immune profiles of OCR-treated patients with MS. (A) SARS-CoV-2-unexposed patients with MS without treatment (n=21), patients with MS treated with OCR (n=57) and healthy controls (HC; n=30) were followed over time after Moderna SARS-CoV-2 mRNA vaccination. Blood or serum samples were collected at specified time points pre-vaccination and post-vaccination. (B) IgG titres to SARS-CoV-2 RBD before vaccination, 28 days after the first, before the second, and 7 and 28 days after the second vaccination. (C) The area under the curve was obtained to quantify the overall anti-RBD IgG antibody responses. (D,E) Age (D) and sex (E) distribution in HC, MS control, and anti-RBD IgG^+^ or anti-RBD IgG^−^ OCR-treated patients with MS. (F–H) The time since the last OCR treatment (F), the concentration of OCR at the day of the first vaccination (G). (H) Correlation plot between the OCR concentration and the time since last OCR treatment at baseline. (I) The number of OCR infusions before the first vaccination (J) FIt-SNE two-dimensional map and cluster identification from FlowSOM analysis of the high-dimensional flow cytometry immune panel, separated across groups and major adaptive immune populations displayed in each projection. (K) Surface expression intensity of indicated markers projected on the FIt-SNE map. (L) The number of circulating neutrophils, monocytes, NK cells, B cells, CD4^+^ T cells, CD8^+^ T cells and γδ T cells per μL of blood on day 0 before the first vaccination. (M) Volcano plot showing the abundance of circulating immune cells fold change of anti-RBD IgG^+^ versus anti-RBD IgG^−^ OCR-treated patients with MS (x-axis) and their Wilcoxon signed-rank test p values (y-axis) at day 0. Statistical significance between groups was determined with Wilcoxon signed-rank test (F, G, I and L) with Bonferroni-Holm multiple comparison correction (C, D and K). Associations in (H) were calculated using Spearman rank correlation. The p values are depicted as *<0.05, **<0.01 and ****<0.0001. AU, arbitrary units; MS, multiple sclerosis; NK, natural killer; ns, not significant; OCR, ocrelizumab; RBD, receptor-binding domain.

The participants underwent a two-dose vaccination schedule with the mRNA-1273 vaccine (Moderna, Cambridge, Massachusetts, USA), administered 42 days apart according to the Dutch national vaccination guidelines. Peripheral blood was collected before vaccination (baseline; day 0) and 7 days after the second vaccination at day 49 (±2 days). In addition, fingerpricks were taken on day 28, day 42 and day 70 post-first vaccination and were used for measuring the antibody responses (figure 1A). Peripheral blood mononuclear cells (PBMCs) were isolated on days 0 and 49 and cryopreserved in liquid nitrogen until required. Nucleocapsid (N) serology and interferon-gamma (IFN-γ) ELISpot after N peptide stimulation assays were conducted at days 0 and 49 to confirm the absence of any prior SARS-CoV-2 infection among the participants. The time between the last OCR infusion and the first vaccination and the number of past infusions were recorded as indicators of OCR therapy.

### RBD and N protein ELISA

SARS-CoV-2 spike RBD and N IgG levels were measured following previously established protocols.^17 18^ RBD and N proteins were generated as described previously.^18^ To analyse the samples, they were diluted 1:1200 and tested for the presence of IgG antibodies targeting the RBD and N. The cut-off value was determined based on the 98th percentile of signal intensities observed in 240 pre-outbreak plasma samples. To quantify the signals, a serially diluted calibrator was employed, comprising pooled convalescent plasma, and this calibrator was included on each plate. The calibrator was assigned an arbitrary value of 100 arbitrary units (AU) per mL (AU/mL). The results were reported as AU/mL, representing a semiquantitative assessment of the concentrations of IgG antibodies. The antibody titres of days 0, 42 and 49 were determined in plasma and for time points 28 and 70 in serum.

### Ocrelizumab ELISA

Plasma samples were collected from the patients with MS receiving OCR treatment and stored at day 0 and day 42 post-first SARS-CoV-2 vaccination, to measure OCR concentration. The measurement was conducted using an ELISA developed by Sanquin Diagnostic Services in Amsterdam, the Netherlands.^16^ This ELISA method involved the use of polyclonal anti-idiotype antibodies specific to OCR, which were used to capture OCR from the serum sample. A biotinylated polyclonal anti-OCR conjugate was then employed to detect the bound OCR, following a sandwich ELISA approach. To generate the anti-idiotype antibodies, rabbits were immunised according to previously described protocols.^19^ Concentrations of OCR in the serum samples were determined by comparing the absorbance with a serially diluted calibrator present on each plate. The lower limit of quantitation for the assay was established as 0.0025 µg/mL.

### Whole blood flow cytometry

Cellular immune populations were assessed using fresh whole blood following the methods described in previous studies.^20 21^ Briefly, 200 µL of fresh whole blood was incubated with two respective antibody panels encompassing 38 unique human immune markers (see online supplemental table 1) for 30 min at room temperature (RT) in the dark. Subsequently, samples were treated with BD FACS Lysing solution (BD Biosciences) for 10 min at RT in the dark, followed by washing and fixation with 1% paraformaldehyde (PFA) for 20 min at 4°C in the dark. After additional washing, samples were resuspended in phosphate-buffered saline (PBS) containing 0.5% bovine serum albumin (BSA) and 2 mM EDTA. Count Bright Plus Absolute Counting Beads (ThermoFisher) were added just before data acquisition to determine absolute cell numbers. The samples were acquired using a BD FACSymphony.

10.1136/jnnp-2023-332224.supp3 Supplementary data

### Computational flow cytometry analysis

The Spectre R package^22^ was used for computational analysis of the data. Initially, the samples were loaded into FlowJo V.10 software, and single cells were gated. Anomalies were identified and removed using the flowAI R package.^23^ Next, an arcsinh transformation was applied, and data points below the detection limit were compressed to reduce noise during the clustering process. To address batch effects, the samples were integrated using reciprocal principal component analysis (rPCA) from the Seurat toolkit for cellular genomics,^24^ which was implemented in Spectre. The rPCA approach projected the data from one batch into the PCA space of another batch. Cells were then paired across datasets using a mutual nearest-neighbour approach,^24^ enabling normalisation of expression levels. A single batch was chosen as the ‘reference’ batch, and each other’s batch was integrated with the reference batch to minimise overall runtime. For subset discovery and high-dimensional FlowSOM data analysis,^25^ the flow cytometry data were analysed using all non-dynamic surface molecules as input. Visualisation of the flow cytometry data was also performed using Flt-SNE in this analysis.^26^ Cluster identities were annotated manually by three individuals independently: monocytes (FSC^int^CD14^+^), neutrophils (FSC^int^SSC^int^CD16^hi^CD10^hi^), natural killer (NK) cells (CD56^+^), B cells (CD19^+^), CD4 T cells (CD3^+^CD4^+^), CD8 T cells (CD3^+^CD8^+^) and gamma-delta T cells (TCRgd^+^CD3^+^), T follicular helper (Tfh) cells (CD3^+^CD4^+^CXCR5^+^), memory T helper (Th) cells (CD3^+^CD4^+^CD45RA^−^CXCR5^−^). Multiple circulating immune populations were then manually annotated in subclusters based on marker expression: B cells (naïve CD27−CD38−, transitional CD38^+^CD10^+^, memory CD27^dim^CD38−, DN-like CD19^hi^CD11c^+^CD21−, activated CD27^dim^CD38−CD11c^+^ B cells, plasmablast CD20−CD27^+^CD38^+^CD138− and plasma cells CD20−CD27^+^CD38^+^CD138^+^), CD4^+^ T cells (central memory (CM) CD45RA−CD27^+^, effector memory (EM) CD45RA−CD27−, EM CD45RA^+^ (EMRA) CD45RA^+^CD27−, stem cell memory CD45RA^+^CD27^+^CD95^+^, naïve CD45RA^+^CD27^+^CD95−), CD8^+^ T cells (CM CD45RA−CD27^+^, EM CD45RA−CD27−, EMRA CD45RA^+^CD27−, stem cell memory CD45RA^+^CD27^+^CD95^+^, naïve CD45RA^+^CD27^+^CD95−). Lymphocytes from the T cell activation/exhaustion panel were clustered and plotted by FIt-SNE and CD4^+^ T cell subsets were manually annotated based on marker expression: Tfh cells (Tfh1 CXCR3^+^CCR6^−^CCR4^−^, Tfh2 CXCR3^−^CCR6^−^CCR4^+^, Tfh17 CXCR3^−^CCR6^+^CCR4^+^), memory Th cells (Th1 CXCR3^+^CCR6^−^CCR4^−^, Th1Th17 CXCR3^+^CCR6^+^CCR4^−^, Th2 CXCR3^−^CCR6^−^CCR4^+^, Th9 CXCR3^−^CCR6^+^CCR4^−^, Th17 CXCR3^−^CCR6^+^CCR4^+^, CXCR3+Th17 CXCR3^+^CCR6^+^CCR4^+^). Additional markers, including HLA-DR and CD38, were used to study the immune dynamics and activation of T cell subsets.

### IFN-γ ELISpot assay

SARS-CoV-2 spike-specific IFN-γ T cell responses were determined by ELISpot assay, which was performed according to the manufacturer’s recommendations and as described before (Mabtech).^27^ On the initial day of the experiment, the PBMC samples were thawed and placed in culture at a temperature of 37°C with a 5% CO_2_ environment. The following day, the cells were counted, and 200 000 cells were plated per well into ELISpot 96-well plates (Mabtech) that were coated with an IFN-γ capture antibody (Mabtech). Specific pools of 15-mer peptides representing two regions of the glycoprotein spike 1 and spike 2 and N (JPT Innovative Peptide Solutions) were introduced to the cultures at a concentration of 1 µg/mL per peptide. Positive controls were stimulated with CD3/CD28 antibodies, while negative dimethyl sulfoxide (DMSO) controls did not receive any stimulation. Two or three biological replicates were taken along per patient and condition. After 16 hours, the cells were discarded, and the plates were stained using the Human IFN-γ ELISpot BASIC kit (ALP) (Mabtech) following the instructions provided by the manufacturer. Subsequently, the spots were counted using an AID plate reader.

### Statistical analyses

Summary statistics, (connected) violin plots, stacked plots, volcano plots and heatmaps were created in R. The ranking metric used in the heatmaps is a score that combines fold change and p value.^28^ Statistical significance was assessed using Mann-Whitney or Wilcoxon signed-rank test using wilcox.test function with Bonferroni-Holm multiple comparison correction in R. Other details, if any, for each experiment are provided in the relevant figure. A detailed description of the multivariate analyses clinical determinants of seroconversion is provided with online supplemental table 2. P values lower than 0.05 were considered statistically significant.

10.1136/jnnp-2023-332224.supp2 Supplementary data

### Data availability

All raw and processed data presented in this study are available at http://flowrepository.org/id/FR-FCM-Z6K4.^29^

## Results

### Number and timing of OCR treatment indicative of seroconversion in patients with MS

The impact of OCR therapy on the efficacy of SARS-CoV-2 mRNA vaccination was studied in patients with MS without (n=21) or with OCR treatment (n=57) and compared with healthy controls (n=30) (figure 1A). On average, MS was diagnosed 11 years earlier (median; IQR 8–19 years). None of the participants had a history of COVID-19-related symptoms. Blood or serum samples were collected at specific time points before and after Moderna SARS-CoV-2 mRNA vaccination (figure 1A). Using a standardised ELISA, SARS-CoV-2 spike RBD-specific IgG antibodies were measured at five time points pre-vaccination and post-vaccination (figure 1B). We identified two groups of OCR-treated patients with MS: those who seroconverted (37% (21 of 57) patients) and those who never seroconverted (63% (36 of 57) patients) (figure 1B). The magnitude of the spike RBD-specific IgG responses in seroconverted OCR-treated patients with MS remained lower across all time points compared with healthy individuals and untreated patients with MS (figure 1B,C). OCR-treated patients (overall 44 years±10; anti-RBD IgG^+^ 45 years±9; anti-RBD IgG^−^ 43 years±11) were slightly younger compared with the MS control group (48±10 years) and healthy controls (48 years±10, not significant) (figure 1D), and females were over-represented in all groups (healthy controls 60% female, MS 76% female, MS-OCR overall 72% female, MS-OCR anti-RBD IgG^+^ 76% female and MS-OCR anti-RBD IgG^−^ 25–69% female) (figure 1E). Further analysis revealed that OCR-treated patients with MS who did not seroconvert after vaccination had a shorter interval since their last OCR infusion, although not significant (figure 1F), accompanied by higher OCR concentration at the day of first vaccination (figure 1G). The two parameters, timing since the last OCR treatment and OCR concentration on the day of the first vaccination, exhibited a significant negative correlation (figure 1H; r=−0.73, p=5.5e-10). Moreover, non-seroconverted patients with MS had received a greater number of OCR infusions before first vaccination (figure 1I). These associations were not influenced by age, gender or disease duration (online supplemental table 2), although time since the last OCR treatment or the OCR concentration was also not significantly associated in a univariate or multivariate logistic regression model. However, our earlier study in a larger MS cohort did find a significant correlation between OCR concentration and patients’ ability to seroconvert.^16^ Together, these findings indicate that the number of treatments and possibly the timing since the last treatment, and overall OCR levels in the blood could be taken into consideration when timing the patient’s SARS-CoV-2 vaccination.

### Deep immune profiling of the SARS-CoV-2 vaccine-induced immune response in OCR-treated patients with MS

To comprehensively analyse the cellular immune response, we employed high-dimensional flow cytometry on 38 unique immune parameters and performed Flt-SNE dimensionality reduction followed by FlowSOM clustering. By evaluating the surface expression intensity of commonly used population-specific markers, the major circulating immune populations were identified and annotated (figure 1J,K). Absolute cell numbers of the seven major immune compartments (neutrophils, monocytes, NK cells, B cells, CD4 T cells, CD8 T cells and γδ T cells) per μL of blood before the first vaccination could not predict which patients would seroconvert upon SARS-CoV-2 vaccination (figure 1L), neither could a more in-depth analysis of 39 uniquely identified circulating immune cell subsets (figure 1M).

### SARS-CoV-2 vaccine-induced B cell responses in seroconverted OCR-treated patients with MS

**Figure 2:**
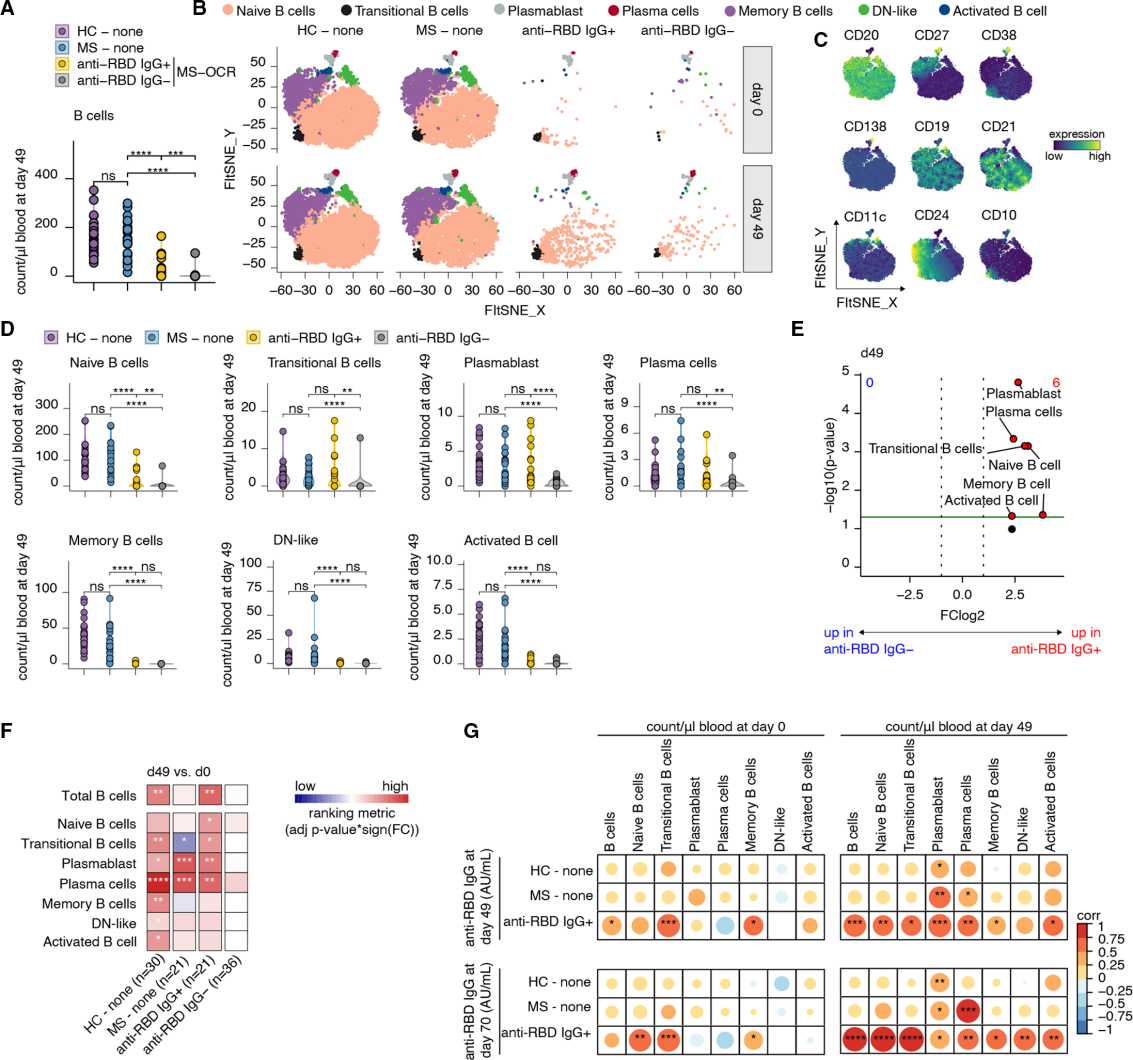
Distinct B cell responses in seroconverted OCR-treated patients with MS after vaccination. (A) The number of circulating B cells per μL of blood on day 49 (7 days after the second vaccination) in healthy control (HC, n=30), untreated MS controls (MS, n=21), seroconverted OCR-treated patients with MS (anti-RBD IgG+, n=21) and non-seroconverted OCR-treated patients with MS (anti-RBD IgG^−^, n=36). (B) FIt-SNE two-dimensional projection and cluster identification of circulating B cell populations from FlowSOM analysis of high-dimensional flow cytometry immune panel. The FIt-SNE projection is separated across groups and time points (day 0 and day 49). (C) The surface expression intensity of indicated markers is projected on the FIt-SNE map. (D) The number of circulating naïve B cells, transitional B cells, plasmablast, plasma cells, memory B cells, double negative (DN)-like B cells and activated B cells per μL of blood 7 days after the second vaccination (day 49). (E) Volcano plot showing the abundance of circulating immune cells fold change of patients with anti-RBD IgG^+^ versus anti-RBD IgG^−^ MS (x-axis) and their Wilcoxon signed-rank test p values (y-axis) at day 49. (F) Heatmap representing changes in B cell population counts per μL blood when comparing baseline (day 0) with day 7 post-second vaccination (day 49) within the different groups, blue indicating a reduction of that specific population between the time points, red an increase and white populations were not affected. (G) Correlations between the number of B cells 7 days after the second vaccination (day 49) and levels of anti-RBD IgG at 7 (day 49; top) or 28 days (day 70; bottom) after the second vaccination. Statistical significance was determined using a Wilcoxon signed-rank test (F,G) with Bonferroni-Holm multiple comparison correction (A and E). Associations in (G) were calculated using Spearman rank correlation. The p values are depicted as *<0.05, **<0.01, ***<0.001 and ****<0.0001. AU, arbitrary units; MS, multiple sclerosis; ns, not significant; OCR, ocrelizumab; RBD, receptor-binding domain.

We performed deep immune profiling at 7 days post-second SARS-CoV-2 vaccination (day 49) to elucidate immunological differences between seroconverted and non-seroconverted OCR-treated individuals. Seroconverted OCR-treated patients with MS displayed a more prominent presence of circulating B cells at this time point (figure 2A). However, the levels of circulating B cells were still lower compared with untreated patients with MS and healthy controls (figure 2A). Additional dimensionality reduction analysis identified distinct circulating B cell compartments, including naïve B cells, transitional B cells, plasmablasts, plasma cells, memory B cells, double negative (DN)-like B cells and activated B cells (figure 2B). These B cell compartments were annotated using established markers (figure 2C).^8^ At baseline, the OCR-treated patients showed a significant decrease in the majority of B cell populations compared with non-treated individuals, regardless of whether they had seroconverted or not (online supplemental file 1). However, the impact of OCR treatment on the circulating plasmablast and plasma cell population at this particular time point was relatively minor. This can be attributed to the fact that as B cells progress into antibody-secreting cells, such as plasmablasts and plasma cells, they gradually lose CD20 surface expression.^30^ At day 49, seroconverted OCR-treated patients with MS demonstrated similar levels of transitional B cells, plasmablasts and plasma cells compared with untreated seroconverted patients with MS, although lower levels of naïve, memory, DN and activated B cells were observed. Interestingly, non-seroconverted OCR-treated patients had significantly lower plasmablast, plasma cells, naïve and transitional B cells compared with seroconverted OCR-treated and untreated patients with MS, while having similar levels of memory, DN and activated B cells (figure 2D,E). Indeed, seroconverted OCR-treated patients with MS demonstrated a similar enlargement of these B cell compartments as compared with those in untreated patients with MS and healthy controls, while these enlargements were absent in non-seroconverting OCR-treated patients with MS (figure 2F). Additionally, a strong positive correlation between the count of naïve, transitional and memory B cell subsets at day 0 and day 49, and the corresponding antibody titres at days 49 and 70, respectively, was observed (figure 2G). This correlation was most pronounced in seroconverted OCR-treated patients with MS (figure 2G). Non-seroconverted OCR-treated patients with MS were not plotted in this correlation, as B cell populations were largely absent in this subgroup. These findings suggest that the dynamics and composition of B cell subsets post-vaccination significantly contribute to the generation and persistence of antibody responses in some OCR-treated patients with MS.

10.1136/jnnp-2023-332224.supp1 Supplementary data

### Robust activation of T cell subsets in seroconverted and non-seroconverted OCR-treated patients with MS

**Figure 3:**
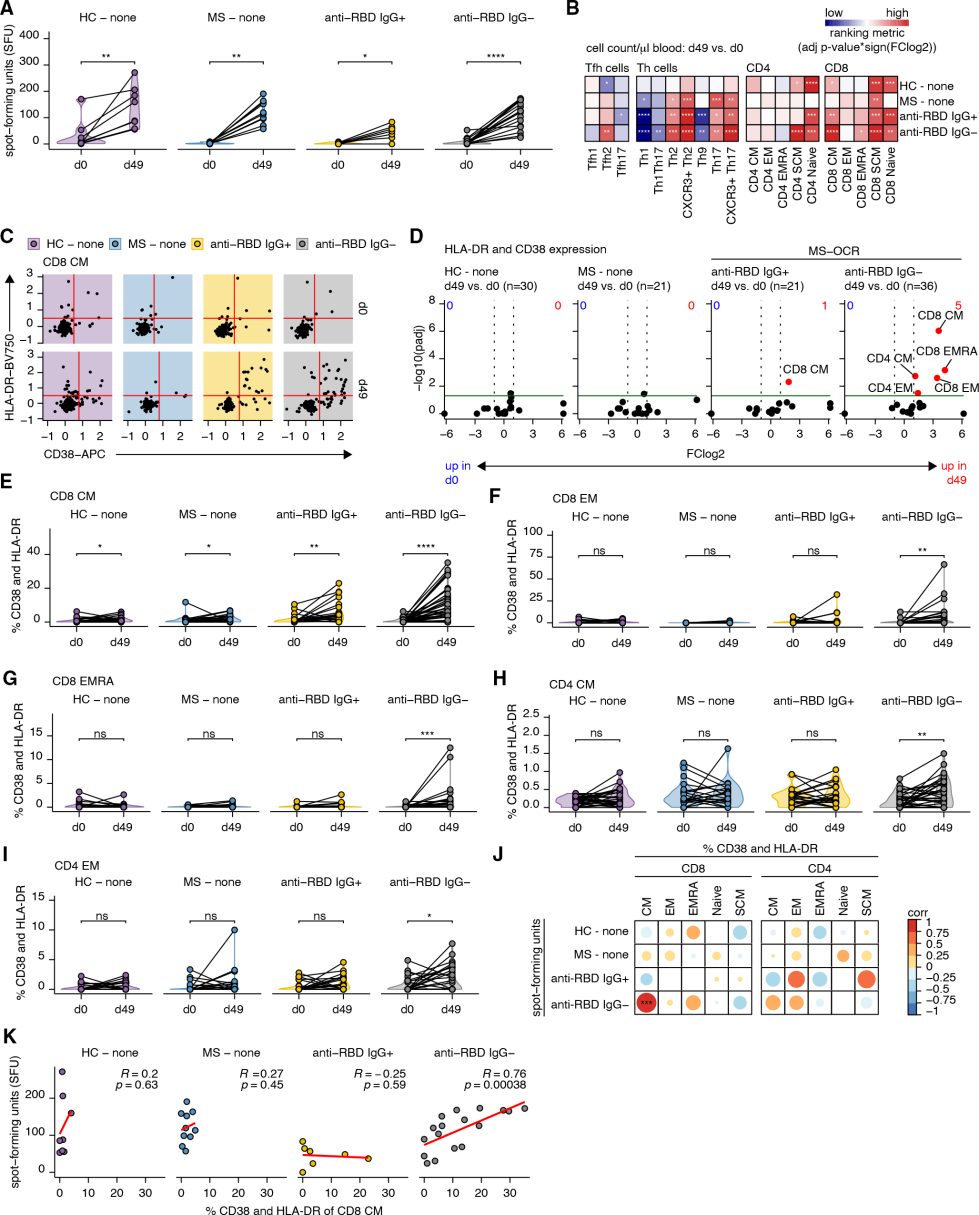
Non-seroconverted OCR-treated patients with MS experience enhanced T cell activation after the second SARS-CoV-2 vaccination. (A) Number of spike-specific IFN-γ–producing T cell spots (SFU) before vaccination and 7 days post-second vaccination are plotted side by side in healthy controls (HC, n=8), untreated MS controls (MS, n=10), seroconverted OCR-treated patients with MS (anti-RBD IgG^+^, n=7) and non-seroconverted OCR-treated patients with MS (anti-RBD IgG^−^, n=17). Results are shown as the average number of SFUs of S1 and S2 together per 2×10^5^ cells after subtracting the SFU of unstimulated wells. (B) Heatmap representing changes in circulating T cell population counts per μL blood when comparing baseline (day 0) with day 7 post-second vaccination (day 49) within the different groups, blue indicating a reduction of that specific population between the time points, red an increase and white populations were not affected (HC, n=30; MS, n=21; anti-RBD IgG^+^, n=21; and anti-RBD IgG^−^, n=36). (C) Representative flow analysis plots showing the expression of HLA-DR and CD38 in CD8 central memory (CM) T cells pre-vaccination and 7 days post-second vaccination. (D) Volcano plot showing the circulating T cell populations expressing both HLA-DR and CD38 fold change of day 49 vs day 0 (x-axis) and their Wilcoxon signed-rank test p values (y-axis). (E–I) Frequency of CD38^+^HLA-DR^+^ CD8^+^ T CM (E), CD8^+^ T effector memory (EM; F), CD8^+^ T EF CD45RA+ (EMRA; G), CD4^+^ T CM (H) and CD4^+^ T EM (I) before (D0) and 7 days after second vaccination (D49). (J) Correlations of the number of spike-specific IFN-γ-producing T cell spots and the frequency of CD38^+^HLA-DR^+^ circulating CD8^+^ and CD4^+^ T cell populations at day 0 and day 49. (K) Correlation plots between the number of spike-specific IFN-γ-producing T cell spots and the frequency of CD38^+^HLA-DR^+^ circulating CD8 CM separated by group. Statistical significance was determined using a Wilcoxon signed-rank test (A (left)–C, E–I) with Bonferroni-Holm multiple comparison correction (A: right). Associations in J and K were calculated using Spearman rank correlation. The p values are depicted as *<0.05, **<0.01, ***<0.001 and ****<0.0001. IFN-γ, interferon-gamma; MS, multiple sclerosis; ns, not significant; OCR, ocrelizumab; RBD, receptor-binding domain; S1, spike 1; S2, spike 2; SCM, stem cell memory; SFU, spot-forming unit; Tfh, T follicular helper; Th, T helper.

In the absence of cross-reactive neutralising antibodies, CD8^+^ T cells form an important second line of protection as they recognise conserved viral peptides in emerging variants^31–34^ and ameliorate disease severity.^20 35–37^ Building upon the observation that some OCR-treated patients with MS displayed no antibody response to the SARS-CoV-2 mRNA vaccination regimen, we conducted a comprehensive analysis of the T cell compartments. Following two vaccinations (day 49), all groups exhibited an increase in IFN-γ spot-forming units, indicative of SARS-CoV-2 spike-specific T cell activation (figure 3A). In accordance with a previous study,^6^ non-seroconverted OCR-treated patients with MS displayed a more substantial T cell activation compared with seroconverted OCR-treated patients with MS (figure 3A). High-dimensional flow cytometry analysis identified different subsets of T cells, including CD4^+^ Tfh cells, CD4^+^ Th cells, and subsets of CD4^+^ and CD8^+^ T cell populations, including naïve, stem cell memory (SCM), EM, terminally differentiated EMRA and CM (online supplemental files 2 and 3). Significant inflation of CD4^+^ Th2 (CXCR3^+^ and CXCR3^−^), Th17 (CXCR3^+^ and CXCR3^−^) and naïve, CM and SCM CD8^+^ T cell compartments and a decrease in CD4^+^ Th1 and Th9 subsets were observed in seroconverted and non-seroconverted OCR-treated patients with MS upon vaccination, while CD4^+^ Tfh2 and SCM responses were uniquely induced in non-seroconverted OCR-treated patients with MS (figure 3B). Healthy controls mainly increased CM and SCM CD8^+^ T cell compartments (figure 3B). Interestingly, untreated patients with MS predominantly increased Th2 (CXCR3^+^ and CXCR3^−^) and Th17 (CXCR3^+^ and CXCR3^−^) subsets (figure 3B). Next, expression of CD38 and HLA-DR was investigated (figure 3C,D), as these markers are known to be specifically upregulated on recently activated T cells and their expression is associated with viral clearance and recovery in healthy individuals^21 38^ and OCR-treated patients with MS.^39^ Several CD8^+^ T CM, EM, EMRA, and CD4^+^ T CM and EM populations were significantly activated in non-seroconverted OCR-treated patients with MS, whereas only CD8^+^ T CM cells demonstrated robust activation in seroconverted OCR-treated patients with MS (figure 3D–I). A significant increase of CD8^+^ T CM activation was also observed in untreated patients with MS and healthy controls, although to a lesser extent without any CD38 and HLA-DR upregulation (figure 3D,E). Correlation analysis revealed a significant link between the activation of CD8^+^ T CM cells and the magnitude of the SARS-CoV-2 spike-specific T cell response, as measured by IFN-γ production upon spike stimulation, in non-seroconverted OCR-treated patients with MS (figure 3J,K). No such correlation was identified in the other groups (figure 3J,K).

These results highlight that the robust and generalised T cell response is uniquely observed in non-seroconverting OCR-treated patients with MS.

## Discussion

We performed in-depth humoral and cellular immune profiling upon two SARS-CoV-2 vaccinations in untreated and OCR-treated patients with MS and were able to stratify OCR-treated patients with MS into those who were seroconverted and those who did not. Our findings revealed that only one-third of OCR-treated patients with MS seroconvert after SARS-CoV-2 mRNA vaccination, although at lower levels compared with untreated patients with MS and healthy individuals. Seroconverted OCR-treated patients with MS had received fewer anti-CD20 treatments and had a longer interval between their last treatment and the initial vaccination, which was associated with reduced levels of circulating OCR as also observed previously.^16 40 41^ Our study is the first to perform in-depth immune profiling on circulating immune cell compartments before initial vaccination, but was unable to identify a factor that could be used to predict a patient’s ability to generate an anti-RBD IgG antibody response. However, seroconverted OCR-treated patients with MS exhibited significantly higher numbers of circulating B cell populations 7 days post-second vaccination compared with those who did not seroconvert. Furthermore, B cell numbers correlated with the antibody response, which was in line with previous observations.^4 40 42^ However, the overall antibody titres and circulating B cell numbers were lower compared with untreated patients with MS and healthy controls, indicating that the B cell response is suboptimal in OCR-treated patients with MS just as expected. Interestingly, our deep immune profiling of the T cell compartments demonstrates that specific subsets of memory CD8^+^ and CD4^+^ T cell compartments are highly activated in non-seroconverting OCR-treated patients with MS, as demonstrated by a strong upregulation of activation markers CD38 and HLA-DR. Although the activation of T cells after anti-CD20 treatment has been observed before,^5–7 27 40 43 44^ our study points to an underappreciated relationship between the dynamics of antibody-producing B cell responses and the dynamics of the overall T cell activation. It also highlights the importance of evaluating CD38 and HLA-DR expression on T cell responses in addition to humoral responses in OCR-treated patients with MS to gain further understanding of the breadth of protective immune response in these patients after vaccination. Overall, our study demonstrates patients treated with B cell-depleting medications are likely to benefit from personalised vaccination schedules which take into account reducing the frequency of OCR treatments and allowing a sufficient interval from the last treatment before vaccination. Patient-optimised vaccination may increase the production of neutralising antibodies against SARS-CoV-2 while maintaining a robust cellular immune response. Our findings are in line with a recently published population pharmacokinetic model which estimated the terminal half-life of OCR in patients with MS at 26 days, while the extent of B cell depletion in the blood was greater in patients with increasing OCR exposures.^45^ The effect of B cell-depleting therapies on longitudinal memory B cell formation and the antibody half-life warrants further investigation.

Indeed, recent studies demonstrated that humoral immune responses can be enhanced in OCR-treated patients, by either decreasing the overall number of OCR treatments and/or increasing the interval between OCR treatment and vaccination; however, the implications for other cellular compartments remain to be investigated.^46–48^ Correlations between clinical parameters and vaccine response provide valuable information for clinicians in managing and tailoring treatments for patients with MS, considering their immunological profiles and disease characteristics. However, this study also has some limitations. The study design was observational, which may introduce biases, and we could not exclude all potential confounding factors due to the limited sample size.^45^ None of the included patients were infected by SARS-CoV-2 during the course of our study. Infections following our final time point were not reported as part of the study; hence, the protective effect of the reduced SARS-CoV-2 RBD-specific antibody titres in seroconverted OCR-treated patients with MS could not be established. We also measured N-specific antibody titres before the first vaccination to verify the patient’s self-reported COVID-19 negative status. It is important to note that the OCR treatment may have negatively affected the patient’s ability to generate N-specific antibodies upon infection, resulting in false negatives. Finally, the functional implications of the observed T cell activation and its potential role in ameliorating disease severity in OCR-treated patients with MS without detectable antibody response require further investigation. Studying the activation status of T cell responses in non-seroconverted patients with MS after OCR therapy in relation to breakthrough infections and/or severity would provide these insights, but may be challenging in terms of achieving sufficient numbers of patients in these patient groups to perform these analyses.

In conclusion, this study provides important insights into the immune response to SARS-CoV-2 vaccination in anti-CD20-treated patients. The findings suggest that while the antibody response may be suboptimal in OCR-treated patients with MS, the induction of broad CD4^+^ and CD8^+^ T cell responses may provide additional protection against severe disease. These valuable insights may be exploited for optimising vaccination strategies in OCR-treated patients.

## Data Availability

Data are available in a public, open access repository. http://flowrepository.org/id/FR-FCM-Z6K4.
