# Supplementary material for: T cell activation markers CD38 and HLA-DR indicative of non-seroconversion in anti-CD20-treated patients with multiple sclerosis following SARS-CoV-2 mRNA vaccination
Source: J Neurol Neurosurg Psychiatry. 2024 Mar 28;95(9):855–64. doi: 10.1136/jnnp-2023-332224 (PMC11347213; doi:10.1136/jnnp-2023-332224)
Supplement: Supplementary data [file jnnp-2023-332224supp003.pdf]

Supplemental Table 1. Whole blood immunophenotyping and CD4 subtyping antibody panels

Panel	Antibody	Clone	Fluorochrome	Dilution	Vendor	Cat no
Immune-phenotype panel	CD45RA	HI100	BUV395	1:400	BD	740298
	CD8	RPA-T8	BUV496	1:1000	BD	612942
	CD14	M5E2	BUV563	1:400	BD	741360
	CD95	DX2	BUV615	1:100	BD	752346
	TCRgd	11F2	BUV661	1:400	BD	750019
	CD16	3G8	BUV737	1:1000	BD	612786
	CD4	SK3	BUV805	1:800	BD	612887
	CD71	M-A712	BV421	1:400	BD	562995
	CD20	2H7	BV480	1:400	BD	566132
	CD3	UCHT1	BV570-P	1:200	Biolegend	300436
	CD11c	B-ly6	BV605	1:100	BD	563929
	CD27	L128	BV650	1:100	BD	563228
	CCR6	11A9	BV711	1:100	BD	563923
	HLA-DR	G46-6	BV750	1:800	BD	746912
	CD45RB	MT4 (6B6)	BV786	1:400	BD	744655
	CD57	NK-1	BB515	1:400	BD	565285
	PD1	EH12.1	BB700	1:400	BD	566460
	CD138	MI15	PE	1:100	BD	552026
	CD10	HI10a	PE-CF594	1:400	BD	562396
	CD56	B159	PE-Cy5	1:100	BD	555517
	CD24	ML5	PE-Cy7	1:200	BD	561646
	CD38	HIT2	APC	1:50	BD	555462
	CD19	SJ25C1	R718	1:400	BD	566946
	CD21	Bu32	APC/FIRE750	1:400	Biolegend	354920
	BD Brilliant Stain buffer plus				BD	566385
CD4/CD8 activation panel	CD45RA	HI100	BUV395	1:600	BD	740298
	CD8	RPA-T8	BUV496	1:1000	BD	612942
	CD27	L128	BUV563	1:400	BD	748705
	CD38	HIT2	BUV615	1:200	BD	751138
	CD137	4B4-1	BUV661	1:200	BD	741642
	CD40-L	TRAP1	BUV737	1:200	BD	748983
	CD4	SK3	BUV805	1:800	BD	612887
	CD127	HIL-7R-M21	BV421	1:200	BD	562436
	CD15S	CSLEX1	BV510	1:800	BD	563529
	CD3	UCHT1	BV570-P	1:200	Biolegend	300436
	CCR7	2-L1-A	BV605	1:100	BD	566754
	TIGIT	741182	BV650	1:400	BD	747840
	CXCR3	1C6/CXCR3	BV711	1:200	BD	563156
	HLA-DR	G46-6	BV750	1:800	BD	746912
	TIM-3	7D3	BV786	1:200	BD	742857
	CCR6	11A9	BB515	1:100	BD	564479
	PD1	EH12.1	BB700	1:400	BD	566460
	ICOS	DX29	PE	1:100	BD	557802
	CCR4	1G1	PE-CF594	1:400	BD	565391
	CTLA-4	BNI3	PE-Cy5	1:400	BD	555854
	CD25	M-A251	PE-Cy7	1:400	BD	557741
	CXCR5	RF8B2	APC-R700	1:200	BD	565191
	CD226	11A8	APC/FIRE750	1:100	Biolegend	338320
	BD Brilliant Stain buffer plus				BD	566385
