# Supplementary material for: T cell activation markers CD38 and HLA-DR indicative of non-seroconversion in anti-CD20-treated patients with multiple sclerosis following SARS-CoV-2 mRNA vaccination
Source: J Neurol Neurosurg Psychiatry. 2024 Mar 28;95(9):855–64. doi: 10.1136/jnnp-2023-332224 (PMC11347213; doi:10.1136/jnnp-2023-332224)
Supplement: Supplementary data [file jnnp-2023-332224supp002.pdf]

	univariate model	multivariate model corrected for age and sex
**time since last OCR infusion**	OR 0.99 95% CI: 0.98-1.00	OR 0.99 95% CI: 0.98-1.00
**OCR concentration at baseline**	OR 1.05 95% CI: 0.99-1.15	OR 1.06 95% CI: 0.99-1.15
**the number of OCR infusions at baseline**	OR 1.65 95% CI: 1.15-2.48	OR 1.69 95% CI: 1.15-2.65
OCR: ocrelizumab, OR: odds ratio		

Supplementary Table 2: multivariate analyses of clinical determinants of seroconversion

Table showing results for multivariate analyses to investigate potential influences of clinical confounders on seroconversion, using a logistic regression model, in MS patients treated with ocrelizumab (OCR). As the IgG responses showed a non-linear distribution (also after logarithmic transformation) this outcome variable did not meet assumptions for a multivariate linear regression model.

Due to the limited sample size of the cohort, the number of potential confounders studied was limited to sex and age, which are well-known determinants of serological responses. To verify this, we studied univariate associations between other potential confounders and seroconversion which were all non-significant in our dataset (BMI p:0.79, disease duration p:0.49, another comorbidity p:0.17).

We repeated the analyses presented in figures on the time since the last OCR infusion (Fig. 1f), the OCR concentration at baseline (Fig. 1g), and the number of OCR infusions at baseline (Fig. 1i) by construction of separate univariate and multivariate logistic regression models (presented with odds ratio (OR) and associated 95% confidence interval). In the univariate logistic regression models, time since last OCR infusion and OCR concentration at baseline did not reach significance while the multivariate models did not show evidence of confounding by age and sex. The number of OCR infusions at baseline was significantly associated with seroconversion and was also not influenced by age and sex.
