# Supplementary material for: T cell activation markers CD38 and HLA-DR indicative of non-seroconversion in anti-CD20-treated patients with multiple sclerosis following SARS-CoV-2 mRNA vaccination
Source: J Neurol Neurosurg Psychiatry. 2024 Mar 28;95(9):855–64. doi: 10.1136/jnnp-2023-332224 (PMC11347213; doi:10.1136/jnnp-2023-332224)
Supplement: Supplementary data [file jnnp-2023-332224supp001.pdf]

Supplementary Figure 1 Circulating immune profiles of OCR-treated MS patients.
(**a**) The number of circulating naïve B-cells, transitional B-cells, plasmablast, plasma cells, memory B-cells, double negative (DN) like B-cells, and activated B-cells per µl of blood at baseline (day 0). Statistical significance was determined using a Wilcoxon signed-rank test with Bonferroni-Holm multiple comparison correction. The *p-values* are depicted as *<0.05, **<0.01, ***<0.001, and ****<0.0001. (**b**) Volcano plot showing the abundance of circulating B-cell population fold change of anti-RBD IgG$^+$ vs. anti-RBD IgG$^-$ MS patients (x-axis) and their Wilcoxon signed-rank test *p-values* (y-axis) at day 0.

Supplementary Figure 2 FIt-SNE two-dimensional (2D) map for CD4$^+$ T-cell subsets based on the CD4 panel. Subclustered FIt-SNE projection of total CD3$^+$ T-cells (**a**), CD4$^+$ T helper (Th) cells (**b**), and CD4$^+$ T follicular helper (Tfh) (**c**). FIt-SNE projection and cluster identification from FlowSOM analysis are indicated on the left and surface expression intensity of the indicated markers is projected on the FIt-SNE map in the middle. Relative expression of T-cell activation markers CD38 and HLA-DR is shown on the right.

Supplementary Figure 3 FIt-SNE two-dimensional (2D) map for CD8$^+$ and CD4$^+$ T-cell subsets based on the immune panel. Subclustered FIt-SNE projection of CD8$^+$ T-cell subsets (**a**) and CD4$^+$ T-cell subsets (**b**). FIt-SNE projection and cluster identification from FlowSOM analysis (left), surface expression intensity of the indicated markers projected on the FIt-SNE map (middle-left). Relative expression of T-cell activation markers CD38 and HLA-DR (middle-right). CD27 and CD45RA expression superimposing the FIt-SNE T-cell subset clusters confirmed with conventional 2D analysis (right).

Verstegen NJM, *et al. J Neurol Neurosurg Psychiatry* 2024; 95:855–864. doi: 10.1136/jnnp-2023-332224
